# Factors Associated with Serological Cure and the Serofast State of HIV-Negative Patients with Primary, Secondary, Latent, and Tertiary Syphilis

**DOI:** 10.1371/journal.pone.0070102

**Published:** 2013-07-23

**Authors:** Man-Li Tong, Li-Rong Lin, Gui-Li Liu, Hui-Lin Zhang, Yan-Li Zeng, Wei-Hong Zheng, Li-Li Liu, Tian-Ci Yang

**Affiliations:** 1 Center of Clinical Laboratory, Zhongshan Hospital, Medical College of Xiamen University, Xiamen, China; 2 Xiamen Zhongshan Hospital, Fujian Medical University, Xiamen, China; 3 Department of Neurology, Zhongshan Hospital, Medical College of Xiamen University, Xiamen, China; Fudan University, China

## Abstract

**Background:**

Some syphilis patients remain in a serologically active state after the recommended therapy. We currently know too little about the characteristics of this serological response.

**Methods:**

We conducted a cohort study using the clinical database from Zhongshan Hospital, Medical College of Xiamen. In total, 1,327 HIV-negative patients with primary, secondary, latent, and tertiary syphilis were enrolled. Bivariate and multivariate analyses were utilised to identify factors associated with a serological cure and serofast state in syphilis patients one year after therapy. Chi-square tests were used to determine the differences in the serological cure rate across different therapy time points.

**Results:**

One year after the recommended therapy, 870 patients achieved a serological cure, and 457 patients (34.4%) remained in the serofast state. The serological cure rate increased only within the first 6 months. The bivariate analysis indicated that male or younger patients had a higher likelihood of a serological cure than female or older patients. Having a baseline titre ≤1∶2 or ≥1∶64 was associated with an increased likelihood of a serological cure. The serological cure rate decreased for the different disease stages in the order of primary, secondary, latent, and tertiary syphilis. A distinction should be drawn between early and late syphilis. The multivariate analysis indicated that a serological cure was significantly associated with the disease phase, gender, age, and baseline rapid plasma reagin (RPR) titre.

**Conclusions:**

The serofast state is common in clinical work. After one year of the recommended therapy, quite a few syphilis patients remained RPR positive. The primary endpoint of the study indicated that disease phase, gender, age and baseline RPR titre were crucial factors associated with a serological cure.

## Introduction

The World Health Organization describes syphilis as a “sexually transmitted infection that can be successfully controlled by public health measures due to the availability of a highly sensitive diagnostic test and a highly effective and affordable treatment.” Nevertheless, syphilis remains a worldwide public health problem [1]–[3]. The global syphilis statistics show that an estimated 10 million new infections still occur each year [4]. The rate of congenital syphilis has been increasing in recent years in China, with an average annual increase of 71.9% [5]. Undoubtedly, we are overoptimistic about the prevention of syphilis, and we still know too little about the disease, especially the serological response after therapy [6].

Parenteral penicillin G has been used for more than 50 years, but no comparative trials have been adequately conducted to guide the selection of an optimal penicillin regimen (i.e., the dose, duration, and preparation) [7]. Meanwhile, the basis for evaluating the therapeutic response remains serological testing [7]. Not all patients achieve serological reversal after the recommended treatment, some patients demonstrate a persistent positive serological reaction that was quite disconcerting for both the physician and patient [8]. It remains unclear whether the persistent positive serological reaction indicates persistent foci of spirochetes or progressive syphilitic lesions or whether it reflects the persistence of reagin in the circulating blood following anti-syphilitic therapy. For these reasons, a discussion about the serological response after the recommended therapy is more than justified.

Serological tests are the most widely used laboratory techniques for diagnosing syphilis and monitoring its post-treatment course [7]. Serological tests can be divided into two categories: nontreponemal and treponemal antibody tests. The titre of nontreponemal antibodies usually correlates with disease activity, and this titre is the basis for evaluating the therapeutic response [9]. The nontreponemal titre usually decline after therapy. In some patients, nontreponemal antibodies can persist for a long time in a range that differs little from the baseline rapid plasma reagin (RPR) titre after the recommended therapy. These antibodies sometimes persist for the lifetime of the patient. In a previous study, clinical trial data demonstrated that after undergoing the recommended therapy, approximately 15% of patients with early syphilis did not exhibit two-dilution declines or two-dilution increases in the nontreponemal antibody titre. These individuals were considered to be in a “serofast state” one year after treatment [10]. Experience has indicated that, for some patients, the nontreponemal antibody test results remain in a tight range one year after the recommended therapy. This response is called the syphilis serofast state [11]–[13] or sero-resistance [14].

There is no generally definition of the serofast state (so-called sero-resistance), but most observers agree that the concept should be based on a chosen span of time, amount of treatment and the change of RPR titre [7], [14], [15]. A fourfold (two dilutions) change in titre is considered necessary to demonstrate a clinically significant difference between two nontreponemal test results that were obtained using the same serologic test [16]. Accordingly, in this study, after one year of recommended therapy, syphilis patients were considered to be in a serofast state if their nontreponemal test remained positive and the titres neither increased nor decreased by at least four-fold (two dilutions). Syphilis patients with persistent or recurrent clinical signs of syphilis and whose nontreponemal antibody titres increased by four-fold or more were considered to exhibit treatment failure or reinfection. Syphilis patients whose clinical manifestations disappeared and whose nontreponemal antibody titres became negative or decreased by four-fold (two dilutions) were regarded as achieving a serological cure. We still know little about the serological response, and the factors that predict the serological response after the recommended therapy among syphilis patients have not yet been thoroughly studied. In our previous study [11], [12], we found that Tp-IgM could be used as a serological marker for relapse and syphilis infection. To further explore the characteristics of the serological response, we investigated the factors associated with serological cure and the serofast state in this cohort study.

## Methods

### Study Population and Ethics Statement

All subjects in the present study were collected from the clinical database of Zhongshan Hospital, Medical College of Xiamen, between June 2005 and May 2010. This study was approved by the Institutional Ethics Committee of Zhongshan Hospital, Medical College of Xiamen University, and was in compliance with the national legislation and the Declaration of Helsinki guidelines. Written patient consent was obtained according to the institutional guidelines.

### Serologic Tests

Laboratory analyses, including RPR (InTec Products, Inc., China) and *Treponema pallidum* particle agglutination (TPPA) (Fujirebio, Tokyo, Japan), were conducted in accordance with the instructions of the manufacturers. All serum samples used in the RPR test were diluted with physiological saline (NaCl 0.9%) at a ratio of 1∶1 to 1∶32 (V/V) to avoid prozone effects and false-negative results. Readings were taken visually and then compared with the negative and positive controls. The titers of the serum samples that were reactive with RPR were quantified using two-fold serial dilutions until the endpoint was determined. Samples with low titers were randomly and blindly checked by other technicians for quality control. Three standard serum samples with low, medium, and high titers from the National Centers for Clinical Laboratory were used as controls for the RPR reaction. The initial dilution of the serum samples for the TPPA reactions was 1∶80.

### Testimony of Diagnosis of Syphilis

In the light of CDC guidelines in USA and European [16], [17], primary syphilis is characterized by an ulcer (chancre), usually with regional lymphadenopathy, and laboratory confirmation is darkfield examination/fluorescent antibody method/PCR to detect *T. pallidum* in lesion exudate, or/and a reactive nontreponemal test and a treponemal test to confirm the diagnosis of syphilis. Secondary syphilis is generally characterised by a maculopapular rash, and the laboratory test confirmation is a reactive nontreponemal test and a treponemal test to confirm the diagnosis of syphilis. For Latent syphilis is asymptomatic with a possible history of infection supported by reactive treponemal tests and nontreponemal test and normal cerebrospinal fluid. Tertiary syphilis was defined as syphilis acquired >1 years previously [17], clinical manifestations (i.e., cardiac or gummatous lesions) and a history of primary, secondary, or latent syphilis, moreover, the laboratory test confirmation by reactive nontreponemal and treponemal test.

### Syphilis Treatment

Parenterally administered penicillin G is the preferred drug for treating all stages of syphilis [18]. The preparation used (i.e., benzathine, aqueous procaine, or aqueous crystalline), the dosage, and the length of treatment depend on the stage and clinical manifestations of the disease. Generally, non–penicillin-allergic participants underwent treatment randomisation to receive benzathine penicillin (2.4 million U by intramuscular injection for early syphilis or 7.2 million units total, administered at 3 doses of 2.4 million units intramuscular injection each at 1-week intervals for late syphilis). Penicillin allergic participants were randomised to receive doxycycline (100 mg taken orally twice daily for 14 days) or azithromycin (2.0 g daily either IM or IV for 10–14 days) [13]. The detailed recommended therapy schedules for different stages of syphilis strictly followed the CDC’s guidelines. Considering that the type of drug used, the dosage, and the route of administration affected the serological cure. Therefore, only patients treated with penicillin were included in our study. After treatment, patients were advised to undergo serum RPR testing every three months for the first year.

### Data Analysis

Statistical analysis was conducted using SPSS version 17.0. Bivariate analysis was utilized to determine the factors associated with the serological cure. The odds ratios (OR) was estimated with 95% confidence intervals (CIs) from the bivariate analysis, and factors with *P*<0.2 were further identified in the multivariate analysis. Adjusted odds ratios (AOR) with 95% confidence intervals (CIs) were also estimated from the regression analysis. Chi-square tests were conducted to identify significant differences across different therapy time points, and *P*<0.05 (two sided) was considered a statistical difference.

## Results

### Characteristics of the Study Population

**Figure 1:**
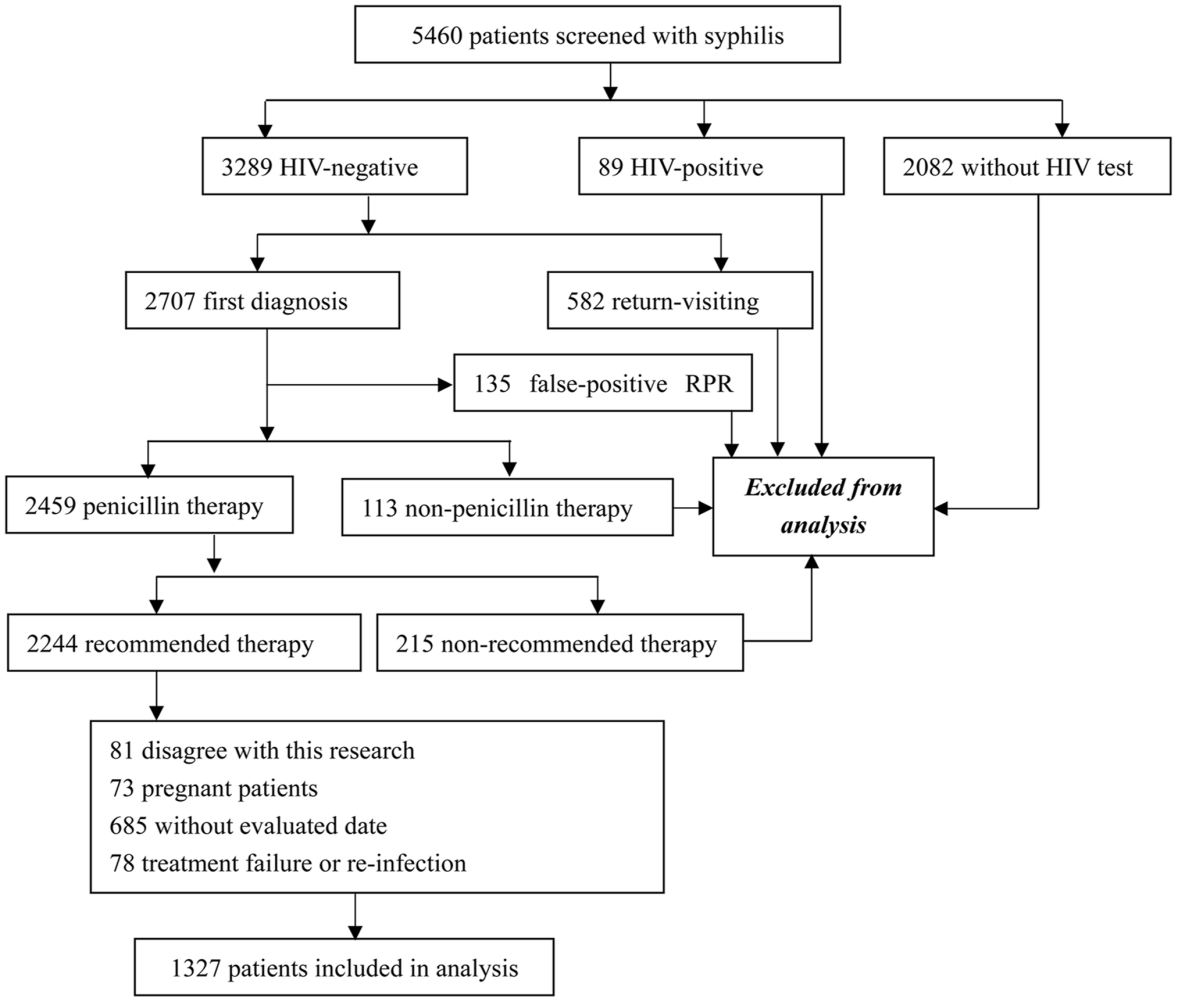
Patient selection criteria used in this study.

A total of 5,460 syphilis patients were found in the Zhongshan Hospital database from June 2005 to May 2010. After excluding the records without documentation of HIV testing and records for HIV-positive or returning patients, 2,707 patients remained. We further excluded patients who did not receive the recommended therapy of penicillin, such as irregular treatment, intermittent treatment, and undertreated therapy. We excluded patients with a false-positive RPR test to avoid the influence on serological response by diseases such as leprosy, systemic lupus erythematosus, HBV, rheumatoid arthritis, thyroid conditions, and respiratory infections. Individuals who were pregnant, experienced treatment failure or reinfection during the course of therapy, had no complement information, or did not agree to participate in our study were also excluded At last, only 1,327 patients were included into our study sample (Figure 1). The mean age was 41.02 years, and 58.9% were men. In this sample, 31.4% patients had latent syphilis, 22% had primary syphilis, 27.1% had secondary syphilis, and 19.5% had tertiary syphilis. One year after treatment, 65.6% exhibited a serological cure, but 34.4% were serofast (Table 1).

**Table 1:** Bivariate analysis of the characteristics of the patients who achieved serological cure.

Characterstic	Serological cure (n = 870)	Serofast (n = 457)	OR	OR(95% CI)	
				Low	Upper
Gender					
Female	312	233	1		
Male	558	224	1.860	1.478	2.341
Age					
<23	57	13	2.023	1.080	3.790
23–29	144	113	0.588	0.434	0.796
30–40	281	152	0.853	0.654	1.112
>40	388	179	1		
Baseline RPR Titer					
1∶1	310	77	2.984	1.979	4.498
1∶2	171	73	1.736	1.134	2.658
1∶4	77	61	0.936	0.586	1.494
1∶8	55	57	0.715	0.437	1.171
1∶16	77	73	0.782	0.495	1.234
1∶32	95	53	1.329	0.832	2.122
≥1∶64	85	63	1		
Phase					
Primary	250	42	1		
Secondary	231	129	0.298	0.195	0.453
Tertiary	140	119	0.196	0.126	0.305
Latent	249	167	0.247	0.161	0.381

* Abbreviations: OR, odds ratios; CI, confidence interval.

### Factors Associated with Serological Response

Our study compared the characteristics of 870 patients who achieved a serological cure with the characteristics of 457 serofast patients at 12 months after therapy. Male patients were approximately 2-fold more likely to achieve a serological cure than female patients. Compared to patients <40 years of age, patients between 23 and 29 years of age had a lower probability of achieving a serological cure, and patients <23 years old were comparatively more likely to achieve a serological cure. Patients with lower or higher baseline RPR titres were easily treated and achieved serological cure, but patients with a baseline RPR titre of 1∶8 or 1∶16 had a decreased likelihood of achieving a cure at 12 months. The serological cure rate for the different disease stages decreased in the order of primary, secondary, latent, and tertiary syphilis (Table 1).

A multivariate analysis was also conducted using all of the significant variables identified above. As shown in Table 2, the results further confirmed that the disease phase, gender, age, and the baseline RPR titre were significantly associated with the likelihood of a serological cure. An age <23 years was associated with a 2-fold greater probability of a serological cure than an age >40 years, and got the highest AOR. Patients with baseline RPR titres of 1∶1 and 1∶2 had high AORs of 2.732 and 2.380, respectively.

**Table 2:** Multivariate analysis of the characteristics of patients who achieved serological cure.

Parameter	AOR	95.0% C.I. for AOR	
		Lower	Upper
Secondary VS Primary	0.372	0.244	0.568
Tertiary VS Primary	0.209	0.133	0.328
Latent VS Primary	0.341	0.223	0.522
Female VS Male	0.594	0.460	0.767
1∶1 VS≥1∶64	2.732	1.772	4.212
1∶2 VS≥1∶64	2.380	1.507	3.760
1∶4 VS≥1∶64	1.438	0.871	2.374
1∶8 VS≥1∶64	0.830	0.489	1.408
1∶16 VS≥1∶64	1.064	0.651	1.737
1∶32 VS≥1∶64	1.617	0.990	2.642
<23 VS >40	2.190	1.133	4.230
23–29 VS >40	0.564	0.401	0.794
30–40 VS >40	0.755	0.566	1.007

* Abbreviations: AOR, adjusted odds ratio.

### Serological Response at Different Time Points after Therapy

**Figure 2:**
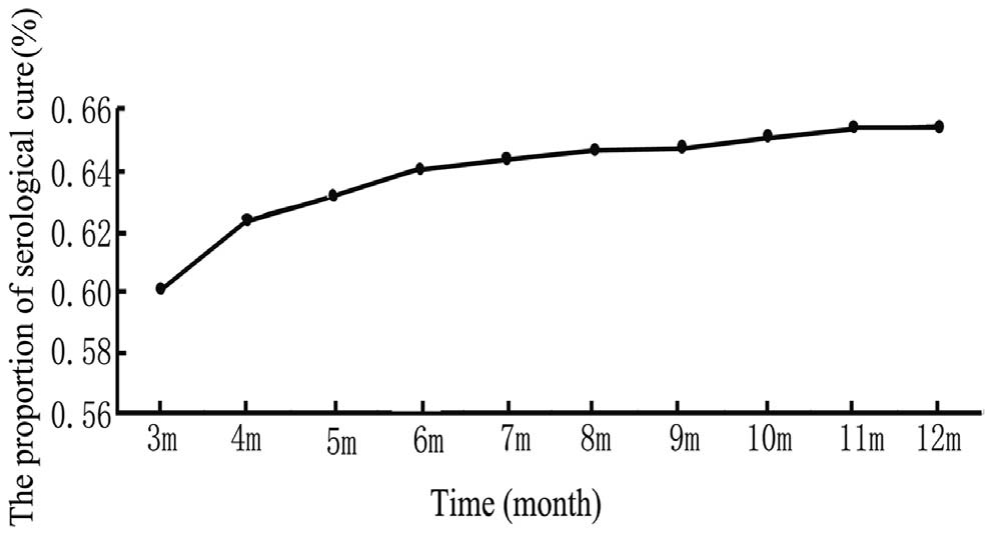
The proportion of the serological cure over time from 3 to 12 months. * Over the first year after therapy, the serological cure rate increased over the first 6 months; for 6–12 months after treatment, the serological cure rate exhibited only a slight increase, and there were no significant differences in the serological cure rate from 6 to 12 months (*X* ^2^ = 0.724, *P* = 0.868).

Because the proportion of patients who achieve a serological cure generally increases with time after treatment, the cure rate varied over time. In our research, the serological cure rate was 59.8% (794/1,327) at 3 months and improved to 64.1% (850/1,327) at 6 months. For 6–12 months after treatment, the serological cure rate exhibited a slight increase (from 64.1% to 65.6%), but there were no significant differences among different time points from 6 to 12 months. The serological cure rate did not change substantially with the time after 6 months (Figure 2).

### Serological Responses for Different Baseline RPR Titres

**Figure 3:**
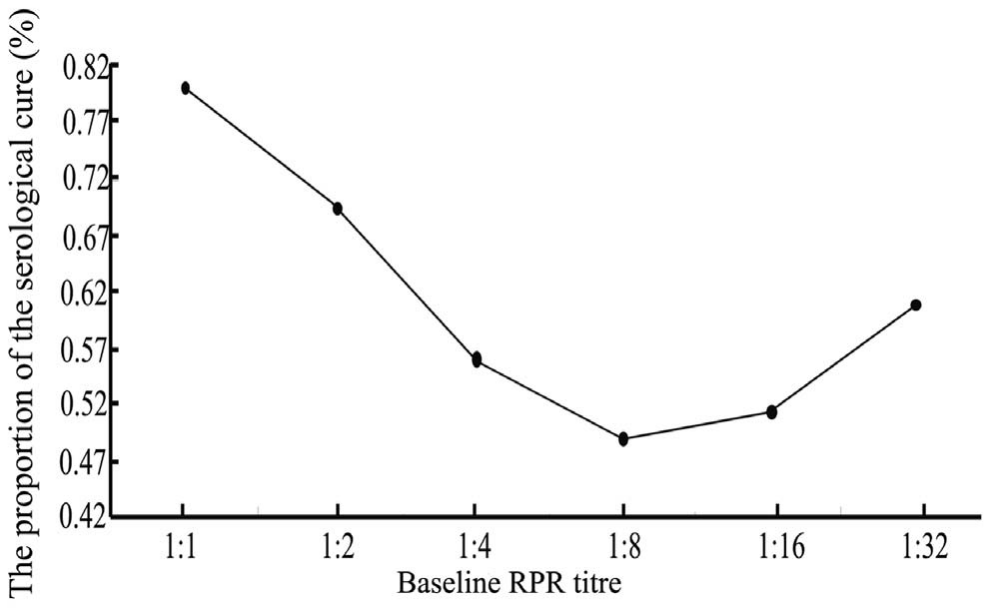
The proportion of serological cure by baseline RPR titre. * The proportion of the serological cure changed with changes in the baseline RPR titre. Overall, the patients with lower or higher baseline RPR titres were more likely to achieve a serological cure (*X* ^2^ = 74.12, *P* < 0.001).

The statistical analysis showed that patients with lower or higher baseline RPR titres were more likely to achieve serological cure. Among the 387 patients with baseline RPR titres of 1∶1, the proportion who achieved serological cure was approximately 80.1%, and among the 272 patients with baseline RPR titres of ≥1∶32, the serological cure rate at 12 months was 60.8%; however, only 49.1% of patients with baseline RPR titres of 1∶8 exhibited a serological cure (Figure 3).

### Difference in Serological Responses between Early Syphilis and Later Syphilis

It is difficult to distinguish early latent syphilis and late latent syphilis. We excluded all the latent syphilis patients and further discussed the difference in the serological cure rate between early syphilis (including primary and secondary syphilis) and late syphilis (including tertiary syphilis). The results indicated that patients with early syphilis had a higher likelihood of a serological cure at 12 months than patients with late syphilis, and the OR was 2.391 (Table 3).

**Table 3:** Comparison of the responses to therapy between early and late syphilis.

Phase	Serological cure (n = 604)	Serofast (n = 286)	OR	P	95.0% C.I.for OR	
					Lower	Upper
Late	140	119	1			
Early	481	171	2.391	<0.001	1.771	3.228

* The serological cure rates of early and late syphilis patients were compared using bivariate analysis. There was a significant difference in the serological cure rate between early and late syphilis patients (*P* <0.001). The OR was used to access the serological cure.

## Discussion

Previous research has discussed the serological response to syphilis treatment [19]–[22]. These studies focused primarily on assessing the effect of HIV infection on the serological response to the treatment of syphilis [19], [20] or on identifying treatment failures or reinfections [22]. To date, only one other paper has reported a systematic evaluation to identify predictors of a serological cure and the serofast state after syphilis treatment [15], which focused only on early syphilis patients, and the sample size was relatively small. In this study, a total of 1,327 cases with different stages of syphilis met the inclusion criteria and were included. Our study was an overall evaluation of the factors that influence the likelihood of achieving a serological cure in patients with different syphilis stages. The results indicate that among the 1,327 syphilis patients who were treated with the recommended therapy, 457 patients remained in a serofast state one year after treatment according to the nontreponemal antibody test, and the prevalence of the serofast state was 34.4%.

What are the factors that influence the likelihood of achieving a serological cure? In this study, using bivariate and multivariate analyses, we found that the disease phase, gender, age, and the baseline RPR titre were associated with a serological cure. The serological cure rate is affected by numerous factors, and we cannot deny the significance of other factors that were not included in the logistic model with multiple variables. A previous study indicated that the type of drug used, the dosage, and the route of administration also affected the serological cure rate [23]. Meanwhile, the relationship between the serofast reaction and neurosyphilis has received a great deal of attention [24]. In our prior study, we analysed the cerebrospinal fluid from 205 patients in the serofast state, 115 of which (56.1%) were diagnosed with neurosyphilis [25]. That study demonstrated the close relationship between the two diseases. Another published study suggested that the serological response was influenced by the body’s immunological function [26].

For many syphilis patients, the serological response varies over time after the recommended therapy. As stated previously, the time point used in the analysis is important when assessing the serological response [15]. Typically, nontreponemal antibody titres should decrease at least fourfold within 6 months after treatment of primary or secondary syphilis and within 12 months after treatment of latent or late infection [21]. In this research, we compared the serological cure rates at different time points after treatment. The results indicate that the proportion of serological cure increased within the first 6 months, but from 6 to 12 months after treatment the serological cure increased only slightly. These findings generally support those of other investigations regarding that the likelihood of a substantial decline in the serofast proportion over time is low. [22]. Considered in this light, it would be appropriate to use a 12-month follow-up standard for syphilis treatment in this research.

In our study, the prevalence of the serofast state in females was 42.8%, which was higher than that in males. This result was different from the result of a previous study that denied this association [15], [27]. Generally, the immune system differs between males and females [28], and the serological response is also influenced by the immune system [19], so gender difference could influence the serological response after treatment. The exact mechanism of this association was still unclear in present research and it needs more detailed investigation. Meanwhile, we found that patients >40 years old had a comparatively lower probability of achieving a serological cure than patients <23 years old. A retrospective analysis found that older age was associated with a lower probability of a serological cure or reduction in the baseline RPR titre [29]. This older population usually demonstrates increasing senescence of the immune system and immunosuppression that would affect the serological response to syphilis therapy [13], [15].

Our findings also generally support those of other investigations regarding the relationship between the baseline RPR titre and the serological response [22]. After therapy, the serological cure rate first decreased for titres from 1∶1 to 1∶8 and then gradually increased as titres increased. A previous study indicated that a high baseline RPR titre signifies a beneficial inflammatory and immune response to *Treponema pallidum* (*Tp)*, which facilitated the clearance of *Tp* [26]. This observation could be explained by an earlier study [30] that demonstrated VDRL-immunised rabbits exhibited partial protection against reinfection with *Tp*. It is conformed that HIV significantly influenced the serological response after treatment [31], [32]. We did not evaluate the effect of HIV in our study because only 5 HIV-positive patients met our inclusion criteria, and this was not appropriate for statistical analysis.

The influence of the type of syphilitic infection at the beginning of treatment on the serological reaction is an infrequently discussed point [8]. Our results indicated that the serological cure decreased in the order of primary, secondary, latent, and tertiary syphilis. There were 29.3% and 26.2% of patients with early syphilis who did not demonstrate a serological cure at 6 months and 12 months after therapy, respectively. A previous study reported that one-fifth of patients treated for early syphilis did not meet the criteria for serological cure at 6 months after therapy [15]. This difference could be properly explained here. The inclusion criteria for our study population differed from the previous study: returning patients were excluded from our study; every enrolled individual had differences in immunological function that would influence the serological response. Another, we thought the different strains pathogenicity were also a key factor. Some previous studies found that the *Tp* subtypes in different regions were not alike, differing in pathogenicity [33]–[36]. Some scholars have reported that the *Tp* subtype in China is different from those in other countries [37], [38].

Our study has several limitations. The eligible patients represented a fraction of the patients diagnosed with syphilis during the study period, raising a concern for a selection bias. Nearly 38.1% (2,082/5,460) of the patients were excluded because they did not have documented HIV test results in our clinical database. Whether the exclusion of these patients affected the outcome is unknown. As a retrospective study, collecting information from medical records previously developed by other professionals is unavoidable but tends to be less accurate. It was difficult to entirely exclude from our research patients with treatment failure or reinfection, which can lead to prolonged seroactivity [19]. The clinical stages of syphilis often overlap, and no concrete indications among the different stages of syphilis exist.

Basic research on serological responses is rare. Our report analyses and discusses the factors associated with a serological cure and the serofast state in syphilis patients after treatment. We hope this discussion proves useful to clinicians and public health workers in identifying patients who may have achieved a serological cure or become serofast, and aids in determining whether serofast patients should undergo a repeated clinical treatment. Given that neurosyphilis is related to the serofast state, cerebral spinal fluid examination should be considered for persons whose nontreponemal antibody titres do not respond appropriately within 6–12 months of therapy or to reinvestigate CSF if the results of a previous examination were normal.
